# Influence of Background Musical Emotions on Attention in Congenital Amusia

**DOI:** 10.3389/fnhum.2020.566841

**Published:** 2021-01-25

**Authors:** Natalia B. Fernandez, Patrik Vuilleumier, Nathalie Gosselin, Isabelle Peretz

**Affiliations:** ^1^Laboratory of Behavioral Neurology and Imaging of Cognition, Department of Fundamental Neuroscience, University of Geneva, Geneva, Switzerland; ^2^Swiss Center of Affective Sciences, Department of Psychology, University of Geneva, Geneva, Switzerland; ^3^International Laboratory for Brain, Music and Sound Research, University of Montreal, Montreal, QC, Canada; ^4^Department of Psychology, University of Montreal, Montreal, QC, Canada

**Keywords:** congenital amusia, emotion, executive control, music exposure, selective attention

## Abstract

Congenital amusia in its most common form is a disorder characterized by a musical pitch processing deficit. Although pitch is involved in conveying emotion in music, the implications for pitch deficits on musical emotion judgements is still under debate. Relatedly, both limited and spared musical emotion recognition was reported in amusia in conditions where emotion cues were not determined by musical mode or dissonance. Additionally, assumed links between musical abilities and visuo-spatial attention processes need further investigation in congenital amusics. Hence, we here test to what extent musical emotions can influence attentional performance. Fifteen congenital amusic adults and fifteen healthy controls matched for age and education were assessed in three attentional conditions: executive control (distractor inhibition), alerting, and orienting (spatial shift) while music expressing either joy, tenderness, sadness, or tension was presented. Visual target detection was in the normal range for both accuracy and response times in the amusic relative to the control participants. Moreover, in both groups, music exposure produced facilitating effects on selective attention that appeared to be driven by the arousal dimension of musical emotional content, with faster correct target detection during joyful compared to sad music. These findings corroborate the idea that pitch processing deficits related to congenital amusia do not impede other cognitive domains, particularly visual attention. Furthermore, our study uncovers an intact influence of music and its emotional content on the attentional abilities of amusic individuals. The results highlight the domain-selectivity of the pitch disorder in congenital amusia, which largely spares the development of visual attention and affective systems.

## Introduction

Music is prevalent in the modern environments of our daily life. Frequently used in concomitance with mental or motor routine activities such as working out, reading a book, or driving a car, music exposure is also present in many public places, such as restaurants or shops. Although music can be distracting (Kämpfe et al., 2011; Silvestrini et al., 2011), it also has the capacity to enhance cognitive or physical activities in various populations, including Alzheimer's and Parkinson's disease patients, elderly with fall risks (Trombetti et al., 2011; Hars et al., 2013), as well as healthy people (Thompson et al., 2001; Trost et al., 2014; Fernandez et al., 2019b). For instance, music exposure can enhance visuo-spatial attention (Rowe et al., 2007; McConnell and Shore, 2011; Trost et al., 2014; Fernandez et al., 2019b), an essential cognitive ability involving allocating processing resources to specific goal-relevant sensory information.

The beneficial effect of music exposure on visuo-spatial attention may be at least partly related to the emotions music conveys. Previous research has demonstrated the importance of affective states in influencing attention allocation (Mitchell and Phillips, 2007; Vanlessen et al., 2016). Notably, positive affect has frequently been associated with a broader scope of attention (Fredrickson, 2004) which might in turn impair selective attention due to reduced selectivity (Rowe et al., 2007). During music exposure, both positive valence and high arousal may play a similar role in enhancing visuo-spatial information processing (McConnell and Shore, 2011; Trost et al., 2014; Fernandez et al., 2019b).

We (Fernandez et al., 2019b) previously investigated the impact of exposure to music evoking different emotions, including joy, tension, tenderness, and sadness, on the deployment of selective attention processes using a classic visuo-spatial attention network test (ANT) (Fan et al., 2002). In line with other results (Trost et al., 2014), we found that when control participants were exposed to highly arousing background music, especially pleasant music, their performance on the test improved, as revealed by faster target detection in the presence of distractors and greater engagement of fronto-parietal areas (Fernandez et al., 2019b) which is associated with top-down attentional control (Corbetta and Shulman, 2002). These findings are consistent with the notion that rhythmic stimuli can stimulate physiological systems including attentional processes, through their beat structure, especially when the target appears in synchrony with the strong beats of the presented music (Escoffier et al., 2010; Bolger et al., 2013; Trost et al., 2014). The effect of music exposure on selective attention resources is probably mediated by an entrainment of brain rhythms and induced changes in emotional state.

Critically, the above-mentioned studies were conducted using a sample from the general population without regard to individual musical abilities. Notably, people are not equal when it comes to music abilities. Although most people develop normal musical skills, 1.5 to 4% of the population (Peretz and Vuvan, 2017) may suffer from a genetic, music-specific neurodevelopment disorder called congenital amusia (Peretz et al., 2002; Hyde and Peretz, 2004). This musical disorder can be separated into two variants: the most common pitch-based form (also referred as “pitch deafness”) and the more recently described time-based form (also referred as “beat deafness”) (Phillips-Silver et al., 2013; Peretz and Vuvan, 2017). In the pitch-based form of congenital amusia, the focus of this study, individuals (*amusics* hereafter) present a dysfunction in the fine-grained processing of the pitch structure of music which plays a fundamental role in developing a normal musical system and in fully experiencing music's subtleties (Peretz, 2016). At the cortical level, congenital amusia is associated with neural anomalies affecting both functional and structural connectivity in fronto-temporal networks of the right hemisphere (Hyde et al., 2011; Peretz, 2016). At the behavioral level, congenital amusia is characterized by a selective impairment in the perception and production of very small (<2 semitones) variations in pitch (Hyde and Peretz, 2004). This pitch deficit can affect several musical tasks, such as singing in tune (Dalla Bella et al., 2009), perceiving dissonance (Ayotte et al., 2002; Cousineau et al., 2012), and recognizing familiar melodies without the aid of lyrics (Ayotte et al., 2002). Although this pitch deficit does not usually appear alongside any other psychoacoustic deficits, many amusics also experience difficulties with rhythm (Ayotte et al., 2002), especially in the presence of pitch variation (Foxton et al., 2006; Phillips-Silver et al., 2013).

While the emotional information conveyed in music and speech largely depends on pitch (among other acoustical features), studies investigating the relationship between emotional sensitivity and pitch deficits present in congenital amusia show contrasting results. Previous studies reported that amusics could still correctly recognize the emotion content expressed by music (Ayotte et al., 2002; Gosselin et al., 2015; Jiang et al., 2017) or by speech prosody (Ayotte et al., 2002; Hutchins et al., 2010) despite their musical impairment. In most cases, amusics were found to rely on alternative acoustic features (e.g., tempo, timbre, or roughness) to correctly distinguish musical emotions (Cousineau et al., 2012; Gosselin et al., 2015; Marin et al., 2015). However, other work observed mild impairments in the discrimination of emotions in music or in speech but with preserved intensity judgements (Lévêque et al., 2018; Pralus et al., 2019), or moderately reduced capacities to discriminate emotional prosody in speech (Thompson et al., 2012; Lolli et al., 2015; Lima et al., 2016) as compared with control participants. In addition to suggesting that pitch processing impacts musical emotion perception, the latter findings could also be explained by recent evidence linking congenital amusia to music-specific disturbances in consciousness. Because of these heterogeneous findings on emotion recognition in amusics and their inability to create conscious representations of pitch, it remains unclear whether amusics may still respond to the presence of affective music exposure in other conditions that rely on more implicit processing (Tillmann et al., 2016; Lévêque et al., 2018; Pralus et al., 2019), or whether their perception of emotional content in music may be dampened by their limited musical resources when attentional demands are focused on other stimuli. In the latter case, amusics' perceptual system might be unable to extract relevant affective cues from music due to disrupted pitch processing, and therefore fail to produce the indirect effects of musical emotions on other cognitive functions, such as those observed in attentional processing. Thus, testing attentional effects of musical emotions in amusics would allow us not only to better characterize the extent of musical deficits in this population, but also to clarify the possible role of pitch processing in driving these effects.

Finally, although congenital amusia deficits are thought to occur without any other (cognitive) impairments, several theories have suggested an intimate connection between music and visual-spatial abilities, particularly linking sound frequency representation with spatial codes (e.g., lower pitch is usually represented lower in space than higher pitch) (Rusconi et al., 2006). In line with this assumption, enhanced attentional processing has been reported in musicians compared to non-musicians (Brochard et al., 2004; Sluming et al., 2007) including better executive control performance measured with an ANT paradigm (Medina and Barraza, 2019). However, the relationship between music abilities and visuo-spatial attention is unclear in musically (or visually) impaired people. While Douglas and Bilkey (2007) linked poor performance on a classic mental rotation task in amusic individuals to their deficits in processing contour components, other studies found preserved performance in a similar rotation task in amusics (Tillmann et al., 2010; Williamson et al., 2011). These divergent findings further motivate our aim to better characterize the relationship between different visuo-spatial attention components and musical capacities in amusia.

Given these gaps in the current literature, we here assess to what extent congenital amusia deficit might interfere with the indirect (implicit) effects of musical emotions on selective attentional processes. To address this question, amusic and control participants performed the Attentional Network task (ANT; Fan et al., 2002) mentioned above while they were exposed to music communicating four emotional expressions (differentially organized along both arousal and valence dimensions), in addition to a silent condition. In one single task, the ANT probes three distinct components of selective visuo-spatial processing, namely, executive control, alerting, and orienting (Posner and Petersen, 1990; Petersen and Posner, 2012). Executive control is characterized as the ability to selectively attend to specific information by filtering out concurrent distractors. Alerting is defined as the ability to maintain a highly reactive state toward sensory stimuli, while orienting involves the ability to change the focus of attention and direct it to a specific feature or location of stimuli. To our knowledge, the present study is the first to evaluate the effects of several properties of music exposure on distinct components of attention in individuals presenting the pitch-based form of congenital amusia, particularly concerning the emotional aspects of music and their underlying arousal and valence dimensions, and also more generally the first to probe for any link between musical and visuo-spatial attention abilities in this population.

## Materials and Methods

### Participants

Fifteen amusic participants meeting the criteria for the pitch-based form of congenital amusia and fifteen controls matched for education level and musical education duration took part in the study. Participants were mainly right-handed. Participants' characteristics are provided in Table 1.

**Table 1:** Participants' characteristics and musical abilities, measured with the Montreal Battery of Evaluation of Amusia (MBEA; Peretz et al., 2003) and the pitch change detection task (Hyde and Peretz, 2004).

**Participants' characteristics**	**Amusic group (*n* = 15)**	**Control group (*n* = 15)**
Gender (*n*)	10F, 5M	11F, 4M
Age (y)	59.3 ± 19.0	57.2 ± 18.7
Handedness (*n*)	13R, 1L, 1A	14R, 1A
Education (y)	17.0 ± 3.4	16.0 ± 3.2
Musical education (y)	1.0 ± 1.0	2.0 ± 2.0
**Musical abilities**		
MBEA–scale (22^a^/30^b^)	17.0 ± 2.0	28.0 ± 1.2
MBEA–melodic composite score (21.4^a^/30^b^)	17.8 ± 2.2	27.6 ± 1.4
25-cents pitch-change detection (%)	32.7 ± 24.7	93.0 ± 7.2

For both amusic and control participants separately, the mean group values are presented with the standard deviation, except for gender information (number of female and male participants). N, number of participants; y, years; F, female; M, male; R, right-handed; L, left-handed; A, Ambidextrous; %percentage correct;

a cut-off value;

b *maximum score*.

Prior to being selected for participation in this study, the participants were tested on their musical abilities with the online test of amusia (Peretz and Vuvan, 2017), the Montreal Battery of Evaluation of Amusia (MBEA; Peretz et al., 2003), and the Pitch-Change Detection task (Hyde and Peretz, 2004), all reliable tools to identify amusic individuals (Vuvan et al., 2018). The online test is composed of three tasks, namely the scale test, the off-beat test, and the out-of-key test. Standard testing with the MBEA comprises the same scale test and additional contour, interval, rhythm, meter, and memory tests (Peretz et al., 2003; Vuvan et al., 2018). A melodic composite score was computed by averaging the scale, contour, and interval values measured with the MBEA. Finally, the pitch-change detection task evaluates the severity of the pitch deficit. This task assesses the participant's accuracy in detecting a pitch change of the fourth tone in a five-tone sequence. Here, the pitch-change detection scores represent the detection accuracy for the smallest pitch change in the task, i.e., a pitch change of a quarter semitone (25 cents), which is the most discriminant change (Hyde and Peretz, 2004). All amusic participants included in the present study scored below cut-off scores for both the scale test (22/30) and the melodic composite test (21.4/30), except for one amusic who scored below cut-off on the scale test but slightly above cut-off on the melodic composite test. These cut-off scores (i.e., 2SD below the mean of a normative sample) were chosen in accordance with latest normative data (Peretz and Vuvan, 2017; Vuvan et al., 2018) and used as inclusion criteria. All control participants presented normal music abilities, while all amusic participants scored below the cut-off, indicating the presence of pitch deficits.

All amusic participants had normal non-verbal reasoning and verbal working memory abilities as assessed by the Matrix Reasoning and the Digit Span tests from the WAIS-III (Wechsler Adult Intelligence Scale; Wechsler et al., 1997). All participants had normal or corrected-to-normal vision, had no hearing deficits, and no psychiatric, neurological, or toxicological history.

Only non-musicians were recruited according to the following criteria: (i) no music education/practice before 10 years old, and (ii) no current/past regular music practice for a duration of over 5 years. All participants provided informed written consent in accordance with the regulations of the Research Ethics Council for the Faculty of Arts and Sciences at the Université de Montréal.

### Auditory Material

Twelve pieces of instrumental classical music validated in previous work (Trost et al., 2012; Fernandez et al., 2019b) were used in the current study and categorized into four emotions, namely joy, tension, tenderness, and sadness. These four emotions are organized along orthogonal dimensions of arousal (i.e., *Relaxing*–*Stimulating*) and valence (i.e., *Unpleasant*–*Pleasant*) (Trost et al., 2012) and represent the major emotion types identified in the Geneva Emotional Music Scale (Zentner et al., 2008). Hence, both joy and tension are typically associated with highly arousing ratings, while sadness and tenderness are defined as low-arousing. Orthogonally, joy and tenderness are categorized as positively valenced, while tension and sadness are negatively valenced. Our musical excerpts comprised three pieces for each of these four categories, and were presented three times each during the experiments. All musical excerpts had a 45-s duration. Acoustic characteristics of music excerpts are presented for the four emotion categories in Table 2.

**Table 2:** Acoustic characteristics of the music excerpts included in the study for the four emotion categories.

**Dimension**	**Name**	**Perceptual characteristics**	**Audio scores**			
			**Joy**	**Tension**	**Tend**	**Sad**
Rhythm	Tempo	Speed at which a piece of music is played	129.83 (45.47)	117.71 (36.35)	118.72 (37.56)	121.24 (36.41)
	Event density	Complexity of the piece; How many musical events (i.e., average of note occurrence) played in one time unit (sec)	3.33 (1.01)	2.71 (1.27)	2.30 (0.84)	1.82 (0.84)
Beat perception	Pulse clarity	How clearly the beat was detectable in the musical piece	0.37 (0.12)	0.34 (0.18)	0.23 (0.08)	0.15 (0.04)
Timbre	Attack numbers	Number of notes onset or pulses in the piece, related to the expressiveness at which the music piece is played	23.25 (5.13)	20.52 (5.78)	17.61 (4.16)	18.25 (5.61)
	Brightness	Sharpness of the sound	0.31 (0.10)	0.33 (0.15)	0.12 (0.04)	0.29 (0.07)
Frequency/Energy-dominant	Inharmonicity	Amount of energy outside an ideal harmony, supposedly reflect the unpleasantness of the sound	0.43 (0.03)	0.41 (0.04)	0.40 (0.02)	0.36 (0.05)
	Loudness	Information of the intensity of the music piece	0.08 (0.03)	0.05 (0.02)	0.08 (0.04)	0.07 (0.05)
	Dissonance	Roughness, and supposedly the unpleasantness the sound	291.23 (222.10)	137.04 (130.12)	244.41 (216.05)	199.64 (266.51)

*Mean audio scores (SD) extracted using MIRtoolbox, are presented for eight acoustic features covering four major dimensions of music in addition to their perceptual characteristics*.

### Experimental Design

#### Attention Network Task

The experimental task took place in a sound-isolated room. Visual stimuli were displayed on a screen at a distance of 50 cm while auditory stimuli were presented binaurally through high-quality headphones (DT 770 pro−250 Ohms, Beyerdynamic) with optimal tolerable loudness determined for each participant. Stimuli presentation and response recording (through a standard keyboard) were controlled using Cogent toolbox (developed by Cogent 2000 and Cogent Graphics) implemented in Matlab 2009b (Mathworks Inc., Natick, MA, USA).

**Figure 1:**
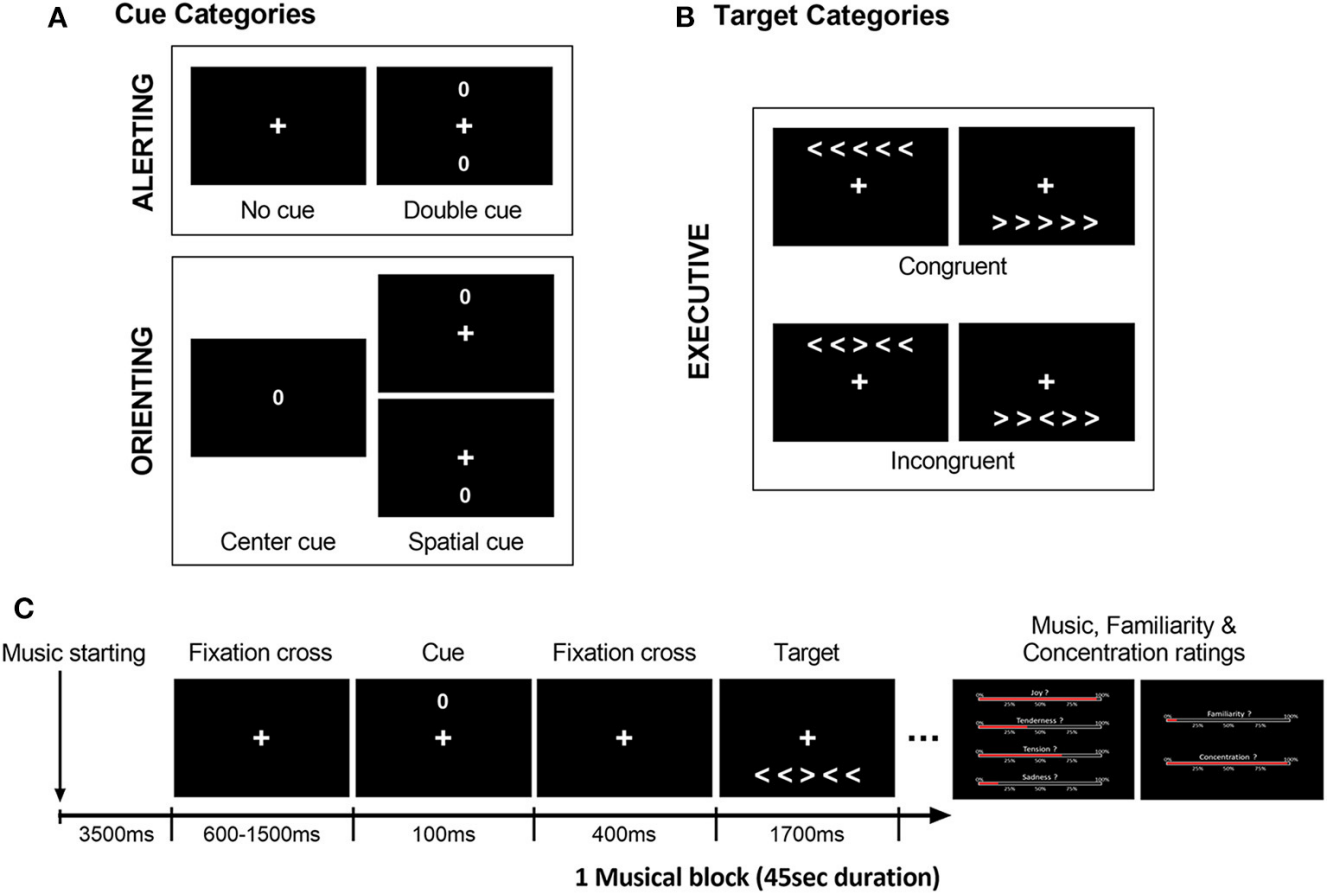
Illustration of the modified ANT design, with distinct **(A)** cue categories and **(B)** target categories used to assess the executive, alerting, and orienting components of attention. **(C)** Example of a typical trial time-course at the beginning of a block (here musical block), with an empty 3,500 ms interval prior to the first trials. All trials began with a cue presentation (spatially invalid cue here), followed by the target display (incongruent condition here). Participants had to indicate the direction of the central arrow of the target (right or left) as fast and as accurately as possible after its presentation (within 1,700 ms max). Music, familiarity and concentration ratings for the preceding musical excerpt were presented after the blocks (i. emotion intensity for joy, tenderness, tension and sadness, ii. familiarity, and iii. concentration).

We used a modified Attention Network Test (ANT; Fan et al., 2002), similar to our previous study in young and older healthy individuals (Fernandez et al., 2019b). In this task, participants are asked to judge the direction of a central arrow (leftward or rightward) considered as the target, presented together with either congruent or incongruent flankers (i.e., distractor arrows with the same or different direction, respectively). This visual display (five arrows including central target) is preceded by one of four types of cues (represented by a zero) at different positions on the screen (Figure 1). These cues correspond to either a central, double, spatial (valid or invalid), or no-cue condition. Based on previous work by Fan et al. (2002), the executive component of attention was assessed by contrasting congruent vs. incongruent conditions regardless of cue type. Alerting and Orienting components were measured by comparing different cue conditions (i.e., double vs. no-cue conditions for alerting; center vs. valid spatial cue conditions for orienting).

Different blocks of the ANT were performed during exposure to the musical pieces from the four different emotional categories as well as during silence to provide a baseline condition. The set of 12 musical excerpts (three pieces for each emotional category) was repeated three times, while the silence condition was presented nine times, leading to a total of 45 blocks (nine for each emotional category or silence condition) of 45 s duration each, and comprising 10 or 11 trials per block. Cue and target types were presented in a pseudo-randomized order and intermixed within the same block. Auditory conditions were also alternated in a pseudo-randomized order between blocks.

The ANT included a total number of 480 trials. As illustrated in Figure 1, for each block, the auditory exposure (music or silence) started 3,500 ms prior to the first visual stimulus appearance. Each trial began with a central fixation cross (duration between 600 and 1,500 ms) followed by one of the four possible cues (100 ms duration, visual angle of 0.91°) and lastly the target display with five arrows (1,700 ms duration). Each cue type (i.e., no-cue, center, double, and spatial cue) was presented in a quarter of the total number of 480 trials. In the no-cue condition (120 trials), only the fixation cross was displayed. In the center cue condition (120 trials), only a single cue circle was displayed at screen center. In the double cue condition (120 trials), two circle cues were presented simultaneously above and below the fixation cross, corresponding to the positions of the target stimuli. In the spatial cue condition (120 trials, regardless of validity), only one cue was presented to indicate either the correct location of the upcoming target (i.e., spatially valid, 60 trials) or the opposite location (i.e., spatially invalid, 60 trials). The cue was followed by an empty 400 ms interval during which only the fixation cross was displayed. The target stimuli consisted of a row of five horizontal arrows, among which the central arrow was the target (visual angle of 5.90° and 1.03°, respectively), randomly presented either above or below the central fixation cross (visual angle of 2.31°). In half of the total of 480 trials (240 trials), the central target arrowhead pointed in the same direction as the flanker arrows [i.e., Congruent condition (Con)], while in the other half of the trials (240 trials), it pointed in the opposite direction [i.e., Incongruent condition (Inc)]. Participants were asked to maintain their gaze directed to the fixation cross and to indicate, as fast and as accurately as possible, the direction of the central arrow after its presentation. Responses were given by pressing a corresponding button on the keyboard (i.e., right or left index for indicating right or left direction, respectively). Buttons presses as well as response times were measured throughout the whole experiment.

This experimental design is similar to our prior study (Fernandez et al., 2019b), with the exception of (i) a central fixation cross that was maintained during the presentation of visual stimuli (Fan et al., 2002), (ii) a slightly smaller visual size of the stimuli, and (iii) an additional silence condition used as a baseline condition.

#### Emotion, Familiarity, and Concentration Ratings

At the end of a subset of the musical blocks of the ANT (i.e., 12 blocks), participants were asked to rate (i) the emotion intensity (i.e., “To what extent the musical excerpt expresses joy, tenderness, tension, and sadness”); (ii) familiarity (i.e., “To what extent were you familiar with this musical piece?”); and (iii) concentration level during the preceding block (i.e., “To what extent were you concentrated on the task?”). Each musical excerpt received four ratings, one for each emotion category along four distinct scales ranging from 0 (= not at all) to 6 (= extremely). Familiarity and concentration ratings were measured using a numerical scale ranging from 0 (= not at all) to 100 (= extremely), which resulted in one single value per musical excerpt. The order of the requested ratings was pseudo-randomized so that each musical excerpt was evaluated once. After a subset of the silence blocks (i.e., three blocks), only concentration level was assessed.

### Data Analysis

All statistical tests were chosen according to the normality of the residuals distribution and the equality of variances in our data, using R Software version 3.2.4 (R., R Development Core Team). The direction of significant interactions was tested using *t*-tests whose resulting *p*–values were adjusted using Bonferroni corrections.

#### Emotion, Familiarity, and Concentration Ratings

First, individual emotion ratings (i.e., emotion intensity) were averaged over the three different musical excerpts for each emotion category (i.e., *joy, tenderness, tension*, and *sadness*). Second, the ability to correctly discriminate musical emotions (i.e., discrimination ratio) was assessed for each emotion category by subtracting the mean intensity scores of the three other categories from the emotion intensity score of a specific category, as used in previous studies of emotion recognition (e.g., Cristinzio et al., 2010). Familiarity ratings were computed for each emotion category by averaging scores for the three different excerpts associated with this emotion. Finally, concentration levels were assessed by averaging the concentration scores obtained for each emotion category (i.e., *joy, tenderness, tension, sadness, and silence*) and each participant.

Three distinct 4 (emotion category) × 2 (group) mixed-model repeated measures ANOVA analyses were used to separately examine emotion intensity, discrimination ratio, and familiarity ratings. The concentration ratings were entered into a 5 (auditory condition) × 2 (group) mixed-model repeated measures ANOVA. The emotion or auditory conditions were considered as within-subject factors, and the group as a between-subjects factor.

#### Attention Network Task

Both the percentage of correct responses (accuracy, AC) and mean reaction times (RT) of correct trials were calculated for each participant and each group separately (*Amusics* and *Controls*), for each of the five auditory conditions (*Joy, Tenderness, Tension, Sadness*, and *Silence*), each target type (*Congruent* and *Incongruent*), and each cue type (*Central, Double, Spatial* and *No-cue*). A trial was considered accurate when participants correctly indicated the direction of the central arrow within the trial time limit (1,700 ms). The three distinct attentional components were separately analyzed according to the specific cues or stimulus combination (Fan et al., 2002; Fernandez et al., 2020). Executive control was assessed by contrasting incongruent vs. congruent arrow conditions regardless of the cue type (i.e., *Con* and *Inc*), while the alerting and orienting components were determined by contrasting distinct cues regardless the target type (i.e., *Double* vs. *No-cue* conditions for alerting; *Center* vs. *Valid Spatial* conditions for orienting).

Because residuals were not normally distributed (preventing the use of parametric statistical tests), AC analyses were performed for each attentional component using paired Wilcoxon rank tests to determine differences between groups and visual conditions (Con vs. Inc; Double vs. No-Cue; Center vs. Valid Spatial). Close-to-ceiling accuracy rates were found in both groups (>95% correct), and no major effect of musical emotion (*p* > 0.3, Wilcoxon rank tests) was found for any component or groups. Consequently, our main analyses and results concerning the influence of music focused on RTs only.

As RT scores revealed normally distributed residuals and equal variances, the effect of music exposure on RT was assessed using three distinct ANOVAs performed, one for each attentional component. Mean RT measures were entered in a separate 2 × 2 × 5 mixed-model repeated-measure ANOVA with trial type (Con and Inc for executive; Double and No-Cue for alerting; Center and Valid Spatial for orienting) and musical emotion category (Joy, Tenderness, Tension, Sadness, and Silence) as within-subject levels and group (Amusics and Controls) as a between-subject factor. Because arousal and valence dimensions are considered as two essential and independent dimensions of all emotion types (Russell, 2003) and thus also describe the functional organization of music-induced emotions (Trost et al., 2012), emotional effects on attention performance and RTs were further assessed by sorting emotion categories into their corresponding arousal and valence dimensions (in line with the 4 categories used in our study). This was achieved using three distinct 2 × 2 × 2 × 2 mixed-model repeated-measure ANOVAs (one for each attentional component) with trial type (Con and Inc for executive; Double and No-Cue for alerting; Center and Valid Spatial for orienting), valence (high and low), and arousal (high and low) as within-subject levels, plus group as a between-subject factor.

The effect of age was determined by entering the mean RT results in three additional 2 × 2 × 5 × 1 mixed-model repeated measure ANCOVAs (one for each attentional component), with the corresponding trial conditions (Con and Inc for executive; Double and No-Cue for alerting; Center and Valid Spatial for orienting) and auditory conditions (Joy, Tenderness, Tension, Sadness, and Silence) as within-subject levels, groups as a between-subject factor, plus age as a covariate.

Finally, Pearson correlation analyses were performed to assess any relationship between musical abilities scores (MBEA scale, melodic composite or pitch-change detection scores) and indices of the three attentional components, namely the executive cost (RT_Inc_−RT_Con_), the alerting efficiency (RT_No Cue_−RT_Double Cue_), and the orienting efficiency (RT_Center Cue_−RT_Spatial valid cue_), calculated following previous work (e.g., Jiang et al., 2011).

Of note, the sample size of the current study was determined by a power analysis using G*Power (version 293 3.1.9.7, Heinrich Heine University). This analysis indicated a probability ≥ 90% to replicate the experimental differences on our attentional task with *n* ≥ 15 based on the effect size (*d* = 1.83) observed in a previous study using the same paradigm in both younger and older individuals (Fernandez et al., 2019b).

## Results

### Attention Network Task Results

**Figure 2:**
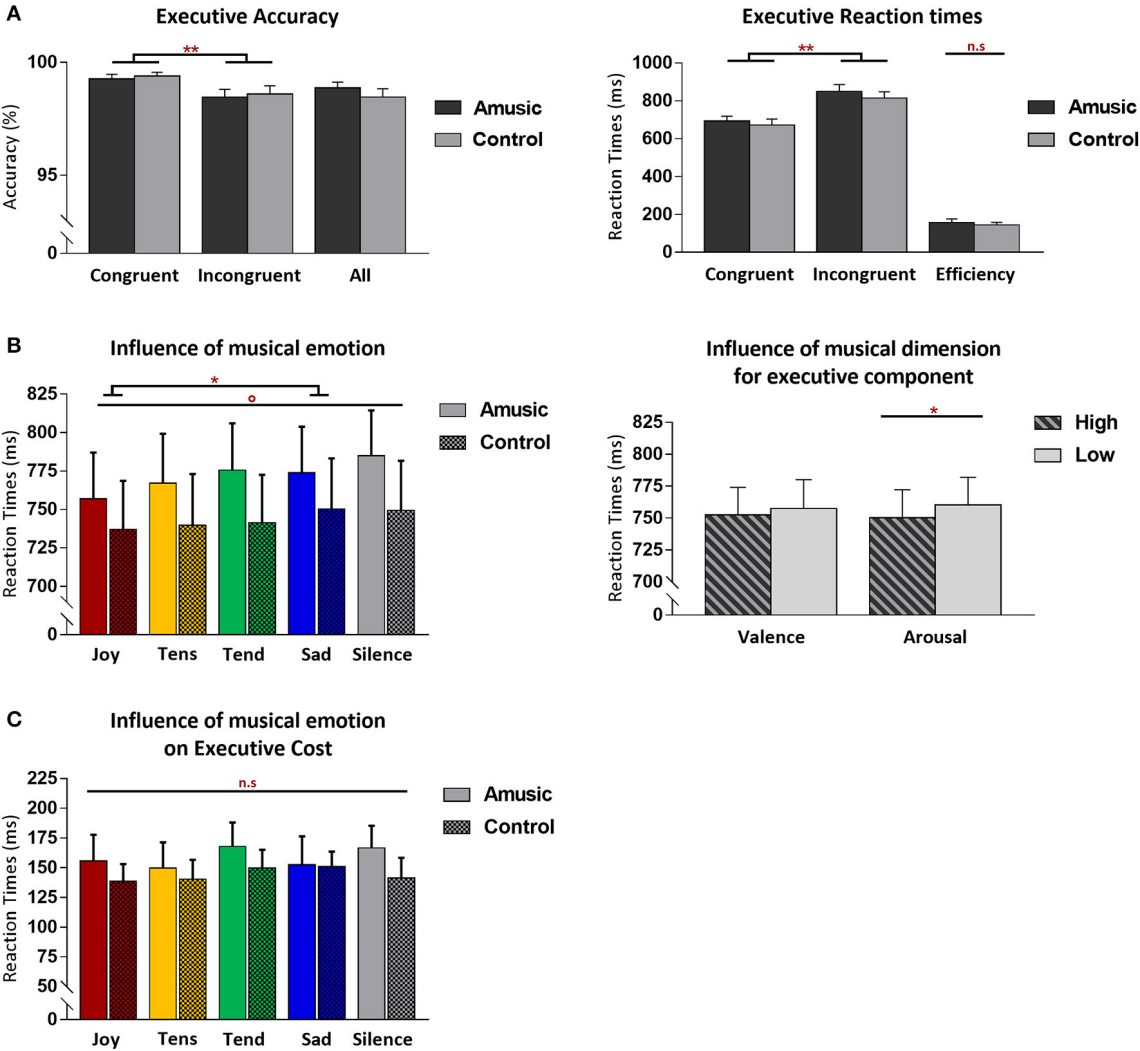
Behavioral results from the Attention Network Task (ANT) for the executive control component of attention, shown for amusic and control participants. **(A)** Mean accuracy (%) (left) and RT (in milliseconds, ms) (right) for congruent and incongruent target conditions. Mean accuracy (%) from both target conditions merged together, as well as interference cost (ms) [(mean RT _Inc_-mean RT _Con_)] are also presented. **(B)** Mean RT (ms) regardless of congruence and group conditions, as a function of the discrete emotion category of music, plus a silence condition (left) or as a function of the valence and arousal dimensions of music (right) during the task. **(C)** Executive cost (ms) as a function of the emotion category of music (plus silence), presented without group distinction. Graphs illustrate standard errors of the mean (SEM) and *p*-values (* or °) with the following meaning: *p < 0.01; **p < 0.001; °*p* < 0.05 without Bonferroni corrections. The n.s. abbreviation indicates non-significant results.

AC (in percentage, %) and RT results (in milliseconds, ms) for each attentional component measured in the ANT are presented in Table 3. Additionally Figure 2 shows more detailed results for the executive control component. Because AC showed no significant effect for music on performance, our main analysis comparing different emotions and different groups focused on RT data.

**Table 3:** Behavioral scores in the Attention Network Task (ANT) for measures assessing executive control, alerting, and orienting components, for both the amusic and control groups.

	**Accuracy [Percentage (SD)]**					**Reaction times [Milliseconds (SD)]**				
**EXECUTIVE NETWORK**										
**A**	**Con**	**Inc**				**Con**	**Inc**	**Cost**		
Amusic	99% (0.7)	98% (1.2)				693 ms (100.0)	850 ms (139.2)	157 ms (76.6)		
Control	99% (0.5)	99% (1.3)				672 ms (126.9)	816 ms (126.9)	144 ms (55.0)		
**B**	**Joy**	**Tens**	**Tend**	**Sad**	**Silence**	**Joy**	**Tens**	**Tend**	**Sad**	**Silence**
Amusic	99% (1.5)	99% (1.6)	99% (1.3)	99% (1.1)	99% (1.2)	757 ms (116.2)	767 ms (124.7)	776 ms (117.9)	774 ms (116.2)	758 ms (113.9)
Control	99% (1.0)	99% (1.1)	99% (1.7)	99% (1.4)	99% (1.3)	737 ms (122.4)	740 ms (128.9)	741 ms (120.5)	750 ms (127.6)	749 ms (124.7)
**ALERTING NETWORK**										
**A**	**No cue**	**Double cue**				**No cue**	**Double cue**	**Efficiency**	
Amusic	99% (0.9)	99% (1.1)				793 ms (119.1)	755 ms (113.2)	37 ms (25.1)		
Control	99% (1.0)	99% (1.2)				750 ms (125.8)	728 ms (132.3)	22 ms (19.8)		
**B**	**Joy**	**Tens**	**Tend**	**Sad**	**Silence**	**Joy**	**Tens**	**Tend**	**Sad**	**Silence**
Amusic	99% (1.7)	98% (2.0)	99% (1.5)	99% (1.3)	100% (0.8)	765 ms (121.9)	764 ms (121.3)	775 ms (117.6)	780 ms (116.3)	786 ms (118.9)
Control	100% (0.9)	99% (1.5)	98% (2.5)	99% (1.3)	99% (1.3)	737 ms (131.5)	739 ms (130.7)	734 ms (125.0)	742 ms (126.9)	743 ms (134.8)
**ORIENTING NETWORK**										
**A**	**Center cue**	**Sp. valid cue**	**Sp. invalid cue**			**Center cue**	**Sp. valid cue**	**Sp. invalid cue**	**Efficiency**	
Amusic	98% (1.3)	99% (1.7)	99% (1.7)			769 ms (115.8)	767 ms (119.1)	771 ms (127.7)	2 ms (20.2)	
Control	99% (1.2)	99% (1.5)	99% (1.1)			738 ms (120.6)	740 ms (124.6)	749 ms (127.1)	−2 ms (25.8)	
**B**	**Joy**	**Tens**	**Tend**	**Sad**	**Silence**	**Joy**	**Tens**	**Tend**	**Sad**	**Silence**
Amusic	99% (2.1)	99% (1.9)	98% (1.9)	99% (1.3)	99% (2.0)	753 ms (115.4)	774 ms (133.0)	773 ms (117.5)	764 ms (114.7)	782 ms (109.6)
Control	99% (1.6)	100% (1.1)	100% (1.0)	99% (1.6)	99% (2.1)	732 ms (116.6)	740 ms (132.9)	743 ms (124.4)	750 ms (132.2)	748 ms (119.0)

*For each attentional component, the upper results (A) display the mean data for each trial type and each cue condition of interest, while the lower results (B) display the mean data for each emotion condition, across the corresponding stimulus and cue conditions. Mean percentage correct [accuracy (%) left panel] and mean reaction times for correct responses [milliseconds (ms), right panel] with standard deviation (SD) values in parentheses. Con, congruent; Inc, incongruent; Sp. Valid Cue, spatial valid cue; Sp. Invalid Cue, spatial invalid cue; Tens, Tension; Tend, Tenderness; Sad, Sadness*.

#### Executive Control Component

Executive control performance was assessed by comparing trials where targets appeared with congruent vs. incongruent flankers. As expected, participants were more accurate (paired Wilcoxon test, *V* = 224.5; *p* < 0.001) and faster [*F*_(1, 28)_ = 156.50; *p* < 0.001] for congruent (*M* = 99%; SD = 0.6; M = 682 ms; SD = 112.6) compared to incongruent trials (*M* = 98%; SD = 1.2; *M* = 833 ms; SD = 130.3). Critically, amusic and control participants did not differ for global executive control performance. Both groups showed similar accuracy scores (*V* = 34; *p* = 0.71), and RT data disclosed no main group effect [*F*_(1, 28)_ = 0.41; *p* = 0.52] nor any group by trial type interaction [*F*_(1, 28)_ = 0.35; *p* = 0.55].

More critically, the influence of music on executive control performance was assessed by comparing RTs between the different auditory background conditions. A first ANOVA performed on RTs across all auditory conditions (four emotion types and silence) and trial types (Con vs. Inc) revealed a main effect of auditory condition [*F*_(4, 112)_ = 3.20; *p* = 0.01] with no group effect [*F*_(4, 112)_ = 0.59; *p* = 0.66] nor any group by trial type interaction [*F*_(4, 112)_ = 0.63; *p* = 0.63]. Joyful music (*M* = 747 ms; SD = 116.9) yielded faster visual target detection than sad music across groups [*t*_(29)_ = −4.06; *p* = 0.003; *M* = 762 ms; SD = 120.2]. An additional ANOVA treating music valence and arousal as separate factors revealed a main effect of arousal [*F*_(1, 28)_ = 8.42; *p* = 0.007] with faster RT during high-arousing (*M* = 750 ms; SD = 120.6) compared to low-arousing music (*M* = 760 ms; SD = 118.2) in both amusics and control participants taken together. No group by arousal interaction was found [*F*_(1, 28)_ = 0.53; *p* = 0.46]. No main effect of valence [*F*_(1, 28)_ = 2.08; *p* = 0.16], nor group by valence interaction was found [*F*_(1, 28)_ = 0.06; *p* = 0.80]. There was no arousal by valence interaction [*F*_(1, 28)_ = 0.25; *p* = 0.61], and no significant group by arousal by valence interaction [*F*_(1, 28)_ = 2.61; *p* = 0.27].

Finally, a significant main effect of age was demonstrated by our covariate analysis, with longer RTs regardless of congruency/trial type and auditory background [*F*_(1, 26)_ = 38.64; *p* < 0.001], reflecting a general age-related slowing in performance which was similar between amusic and control participants [*F*_(1, 26)_ = 2.63; *p* = 0.11]. Specific correlation analyses revealed no association between the executive cost (RT_Inc_-RT_Con_) and musical abilities in amusics, assessed with MBEA scale [*r*_(13)_ = 0.37, *p* = 0.17], melodic composite [*r*_(13)_ = 0.09, *p* = 0.74], and pitch-change detection scores [*r*_(13)_ = −0.07, *p* = 0.78]. No such association was found either in control participants [MBEA scale: *r*_(12)_ = −0.33, *p* = 0.24; melodic composite: *r*_(12)_ = −0.15, *p* = 0.60; pitch-change detection score: *r*_(12)_ = 0.42, *p* = 0.13].

#### Alerting Component

The alerting component of attention was assessed by comparing trials where targets were preceded by double (non-informative) cues vs. no cues. No significant difference in AC was found between the two cue types (paired Wilcoxon test, *V* = 106; *p* = 0.51) or between the two groups (*V* = 115; *p* = 0.93). For RTs, results showed a main effect of cue type [*F*_(1, 28)_ = 51.49; *p* < 0.001] with slower RTs with no-cue (*M* = 771 ms; SD = 122.3) than with double cues (*M* = 742 ms; SD = 121.8). No group effect emerged [*F*_(1, 28)_ = 0.61; *p* = 0.43] nor any group by cue type interaction [*F*_(1, 28)_ = 3.46; *p* = 0.07].

Again, music effects were examined by ANOVAs on RTs measured in different auditory exposure conditions. We found no significant influence of auditory background on alerting for both groups (*p* > 0.04). There was no group by auditory background interaction implicating emotion category [*F*_(1, 28)_ = 0.67; *p* = 0.61], valence [*F*_(1, 28)_ = 0.09; *p* = 0.76], or arousal [*F*_(1, 28)_ = 2.70; *p* = 0.11].

Finally, the age covariate also revealed a main effect on RTs across the different alerting cue types [*F*_(1, 26)_ = 28.99; *p* < 0.001], without group distinction [*F*_(1, 26)_ = 2.19; *p* = 0.15]. None of the two groups showed any correlation of alerting efficiency (RT_No Cue_−RT_Double Cue_) with musical abilities, namely MBEA scale [amusic: *r*_(13)_ = 0.02, *p* = 0.92; control: *r*_(12)_ = 0.11, *p* = 0.70], melodic composite [amusic: *r*_(13)_ = 0.18, *p* = 0.51; control: *r*_(12)_ = −0.59, *p* = 0.02, with did not survive multiple comparisons correction], or pitch-change detection scores [amusic: *r*_(13)_ = −0.27, *p* = 0.31; control: *r*_(12)_ = −0.12, *p* = 0.65].

#### Orienting Component

Finally, orienting effects were probed by comparing performance on trials where targets were preceded by valid spatial cues vs. center cues. AC showed no difference between the two cue types (paired Wilcoxon test, *V* = 67; *p* = 0.16) or between the two groups (*V* = 75; *p* = 0.12). Likewise, RTs showed no effect of cue type [*F*_(1, 28)_ = 0.01; *p* = 0.90], no effect of group [*F*_(1, 28)_ = 0.42; *p* = 0.52], and no group by cue type interaction [*F*_(1, 28)_ = 0.38; *p* = 0.54].

The effects of auditory exposure on RTs were not significant in terms of either emotion category [*F*_(4, 112)_ = 0.61; *p* = 0.65], valence [*F*_(4, 112)_ = 0.47; *p* = 0.49], or arousal [*F*_(4, 112)_ = 0.002; *p* = 0.96], in both amusics and control participants taken together. Although there was a significant interaction between emotion category and cue type [*F*_(4, 112)_ = 2.59; *p* = 0.04], none of the pairwise *post hoc* comparisons survive multiple-comparison correction. The triple interaction emotion category by cue type by group was not significant [*F*_(4, 112)_ = 0.95; *p* = 0.43].

Finally, the age covariate again showed a main effect on RTs [*F*_(1, 26)_ = 41.56; *p* < 0.001] across the two different orienting conditions, without any group distinction [*F*_(1, 26)_ = 1.99; *p* = 0.16]. No correlation was found between orienting efficiency (RT_Center Cue_ −RT_Spatial valid cue_) and musical abilities for amusics: MBEA scale [*r*_(13)_ = −0.08, *p* = 0.76], melodic composite [*r*_(13)_ = −0.12, *p* = 0.64], or pitch-change detection scores [*r*_(13)_ = 1.5, *p* = 0.58]; or for control participants [MBEA scale: *r*_(12)_ = −0.25, *p* = 0.38; melodic composite: *r*_(12)_ = −0.30, *p* = 0.28; pitch-change detection score: *r*_(12)_ = 0.25, *p* = 0.38].

### Emotion, Familiarity, and Concentration Ratings

**Figure 3:**
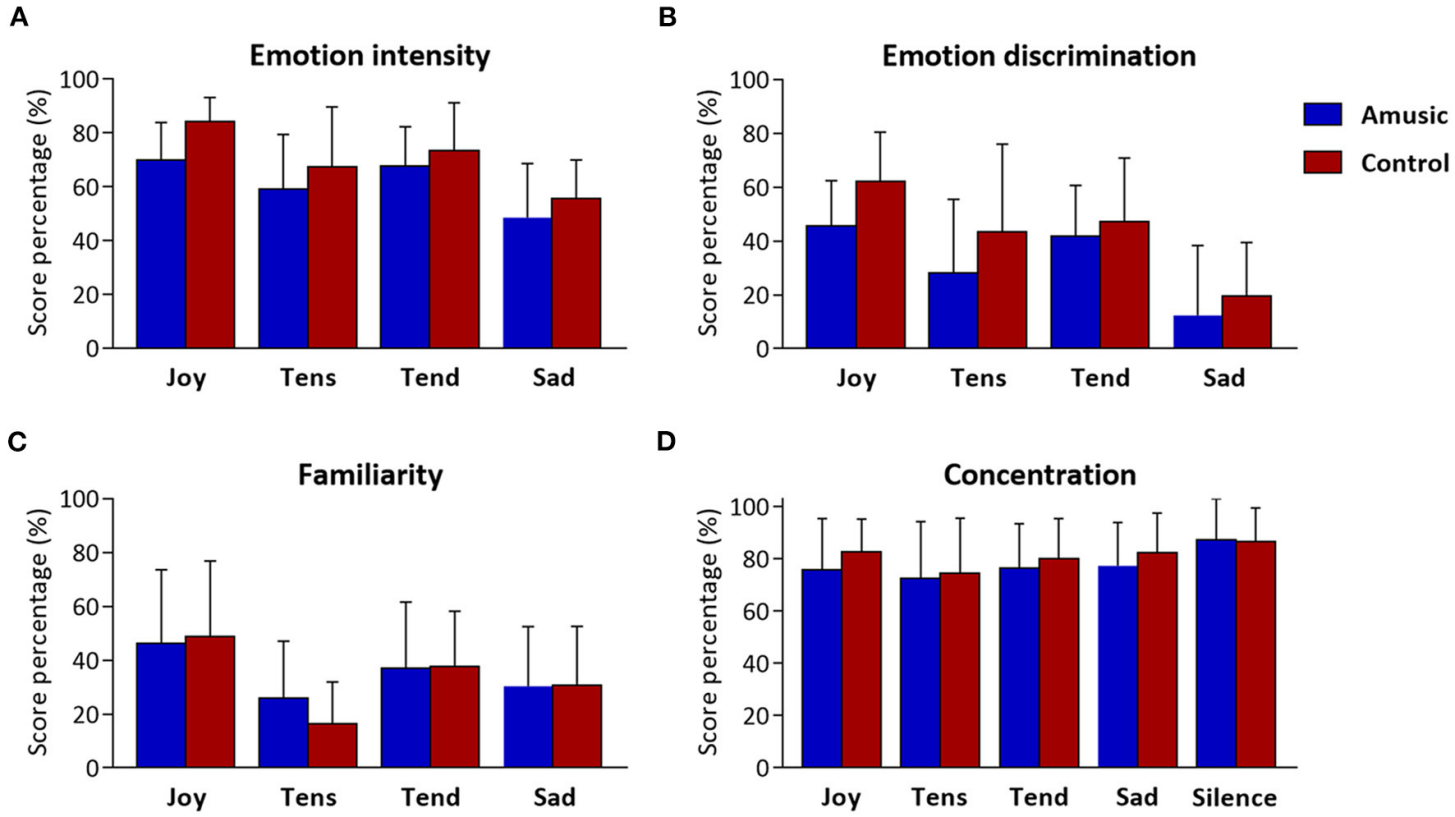
Music and concentration ratings obtained at the end of musical blocks. Scores resulting from **(A)** emotion intensity, **(B)** emotion discrimination (i.e., discrimination ratio), and **(C)** familiarity are presented as percentages (%) for each of the four emotion categories (i.e., Joy, Tension, Tenderness, and Sadness), for amusic and control participants separately. Concentration scores **(D)** are presented (%) for each of the five auditory conditions (including silence) for the two groups separately.

The ratings obtained for emotion intensity, emotion discrimination, familiarity, and concentration ratings are presented in Table 4 and Figure 3. As can be seen, amusics generally judged the emotions expressed by music as less intense than controls did [*F*_(1, 28)_ = 4.48; *p* = 0.04]. In both groups, a main effect of emotion category [*F*_(3, 84)_ = 15.92; *p* < 0.001] showed that joy, tense, and tender music pieces were judged as more intense than sad music. No group by emotion interaction was found [*F*_(3, 84)_ = 0.46; *p* = 0.71]. Emotion discrimination (i.e., relative ratio of ratings for the expressed emotion subtracted by the average ratings given to the other three emotions) showed a main effect of emotion category [*F*_(3, 84)_ = 21.33; *p* < 0.001] with a lower discrimination ratio for sad (*M* = 16%; SD = 23.04) compared to joyful [*t*_(29)_ = −7.55; *p* < 0.001; *M* = 54%; SD = 19.29], tense [*t*_(29)_ = −3.45; *p* = 0.006; *M* = 36%; SD = 30.51], and tender music [*t*_(29)_ = −6.23; *p* < 0.001; *M* = 45%; SD = 21.16]. There was no group effect [*F*_(1, 28)_ = 3.25; *p* = 0.08], nor group by emotion interaction [*F*_(3, 84)_ = 0.64; *p* = 0.58]. Hence, emotion categorization was comparable between amusics and controls.

**Table 4:** Music and Concentration ratings for both amusic and control participants separately.

**Music and concentration ratings**				
	**Emotion intensity**	**Emotion discrimination**	**Familiarity**	**Concentration**
**Joy (V+/A+)**				
Amusic	70% (13.9)	46% (16.9)	46% (27.4)	76% (19.5)
Control	84% (9.1)	62% (18.4)	49% (28.2)	83% (12.6)
**Tension (V–/A+)**				
Amusic	59% (20.1)	28% (27.2)	26% (21.2)	72% (21.8)
Control	67% (22.4)	43% (32.6)	16% (15.6)	74% (21.2)
**Tenderness (V+/A–)**				
Amusic	68% (14.6)	42% (18.8)	37% (24.7)	76% (16.8)
Control	73% (17.8)	47% (23.6)	38% (20.5)	80% (15.2)
**Sadness (V–/A–)**				
Amusic	48% (20.2)	12% (25.9)	30% (22.1)	77% (16.5)
Control	56% (14.4)	19% (20.1)	31% (22.0)	82% (15.2)
**Silence**				
Amusic	NA	NA	NA	87% (15.8)
Control	NA	NA	NA	87% (12.7)

*Music ratings included emotion intensity, emotion discrimination (i.e., discrimination ratio), and familiarity, recorded for each excerpt from the four emotion categories, while concentration ratings were computed for all five auditory conditions (music and silence). Mean values of maximum scores on a continuous scale (from 0 to 100) are presented (in percentage, %) with standard deviation (SD) values in parentheses. NA, Not applicable; V^+^, positive valence; V^−^, negative valence; A^+^, positive arousal; A^−^, negative arousal*.

Familiarity ratings were low for all musical pieces (values < 50), but joyful music was generally rated as more familiar than other emotion categories [*F*_(3, 84)_ = 19.90; *p* < 0.001]. Importantly, there was no group effect [*F*_(1, 28)_ = 0.04; *p* = 0.83], nor group by emotion interaction [*F*_(3, 84)_ = 1.20; *p* = 0.31].

Finally, as expected, subjective concentration ratings revealed a main effect of auditory condition [*F*_(4, 112)_ = 5.70; *p* < 0.001], showing higher concentration ratings during silence than during music exposure conditions [*t*_(29)_ > 2.78; *p* < 0.03], but there was no significant difference between groups [*F*_(4, 112)_ = 0.44; *p* = 0.51], nor an auditory condition by group interaction [*F*_(4, 112)_ = 0.45; *p* = 0.76].

## Discussion

The present study investigated to what extent pitch deficits characterizing the most common form of congenital amusia could influence indirect effects of music exposure on attentional performance. Our results show that major visuo-spatial attention components are preserved in individuals with congenital amusia. Furthermore, and more critically, amusics are still sensitive (just as the general population is) to pleasant and arousing music while performing a visuo-spatial attentional task.

### Impact of Amusia on Emotion Processing

Although emotions conveyed by music were evaluated as less intense by the amusic participants, these individuals categorized the emotions expressed by different pieces with similar accuracy to the control participants. This result is in line with the notion that discrimination and intensity perception might be dissociated in emotion processing (Hirel et al., 2014). Our finding is also consistent with previous work that highlighted relatively spared music-related emotional judgements in congenital amusia (Ayotte et al., 2002; Gosselin et al., 2015). However, the results diverge from other work where poorer emotion recognition was found in amusics as compared to controls for both speech (Thompson et al., 2012; Lolli et al., 2015; Lima et al., 2016) and music (Lévêque et al., 2018). In the latter study, the authors suggested that such a discrepancy could have resulted from the use of orchestral music (Lévêque et al., 2018). Specifically, orchestral music might be more sensitive to subtle amusic deficits during an emotion discrimination task in comparison to the piano music excerpts, like those used in our previous studies (Ayotte et al., 2002; Gosselin et al., 2015). However, in this study, we used orchestral music as well as piano and string music excerpts. Interestingly, more recent work reported deficits in explicit emotion processing in amusics for both music and speech when using a forced-choice method (i.e., choosing a specific emotion among given categories) (Thompson et al., 2012; Lévêque et al., 2018; Pralus et al., 2019), while relatively comparable implicit processing abilities of musical emotions and emotional prosody were observed using indirect investigation methods (e.g., intensity ratings of emotions) (Tillmann et al., 2016; Lévêque et al., 2018; Pralus et al., 2019). Here we employed a free scale method for both intensity and discrimination measures, but these judgments could have engaged participants' internal representations of musical emotions differently compared to explicit emotion categorization tasks with forced-choice labels that require conscious retrieval (Cleeremans and Jiménez, 2002). While discrimination judgments may rely on more strategic access to categorical knowledge based on discrete cues, intensity judgements may involve other perceptual abilities operating on more continuous sensory dimensions. Future work should help further disentangle the complex relationships between musical emotion recognition, auditory complexity, and both explicit/implicit investigation methods in the amusic population.

We also note that all participants had more difficulties correctly categorizing sadness compared to other emotions, and had more success categorizing joy, as found previously (Gosselin et al., 2015; Lévêque et al., 2018). In everyday life, sadness is typically associated with unpleasant feelings. However, in music it is often associated either with pleasantness (Kawakami et al., 2013) or with complex emotions, namely simultaneous positive and negative feelings (Juslin et al., 2014; Sachs et al., 2015), as is similarly reported for nostalgia (Barrett et al., 2010; Trost et al., 2012; Schindler et al., 2017). Such expressions of mixed emotions could be a factor behind the lower discrimination ratios and lower intensity scores observed here. Most importantly, all the differential effects of musical emotion on the ratings were similar between our amusic and control participants. Therefore, the results indicate that amusics do have a fairly average capacity to respond to various musical emotions despite a deficit in pitch processing. This is consistent with a possible dissociation between emotional and perceptual components in the processing of music (Gosselin et al., 2015).

### Impact of Amusia on Attentional Processing

Overall, behavioral performance in the attentional task fully accorded with the literature and showed that the ANT paradigm was effective in assessing visual attention. All participants showed sensitivity to conflicting distractor information when detecting a visual target, with more errors and longer RTs on incongruent compared to congruent trials (Casey et al., 2000; Fan et al., 2005; Fernandez et al., 2019b). They were also faster during trials for which they were temporally warned about the imminent target (i.e., double cue) in comparison to no-cue trials, showing benefits of phasic alertness (Fan et al., 2002; Finucane et al., 2010; McConnell and Shore, 2011). Unexpectedly, behavioral results associated with the orienting component of the ANT did not show any modulation of accuracy or RTs (i.e., for center compared to spatially valid cues), indicating no significant beneficial effect of shifting the attentional focus toward the target location prior to its presentation, contrary to what is typically reported in the literature (Fan et al., 2005; Finucane et al., 2010; McConnell and Shore, 2011). This lack of orienting effect may be caused by an inadequate interval between cue and target displays, or by insufficient spatial preparation following the presentation of the visual cue [perhaps due a 50% validity contingency used here, compared to 100% validity contingency in the original Fan's ANT (Fan et al., 2002)]. Nevertheless, other well-established effects were replicated and accompanied by a significant, age-related slowing of attentional performance, as consistently reported in the literature, particularly for executive control (Zhu et al., 2010; Fernandez et al., 2019a,b). Overall, therefore, our findings converge with previous work on attention and validate our modified ANT task ensuring its sensitivity to assessing congenital amusic individuals in the presence or absence of music exposure.

Critically, our findings for the three distinct attentional components showed comparable accuracy and RT performance between amusic and control participants across all conditions. These findings were confirmed by further correlation analyses revealing that the severity of musical deficits, measured with the MBEA scale, melodic composite, and pitch-change detection, did not predict attentional performance in any of the three attentional components. This spared performance in congenital amusia stands in sharp contrast with executive control deficits documented in several populations, including the elderly (Zhu et al., 2010; Fernandez et al., 2019a) and patients with visual attentional developmental disorders (e.g., ADHD) (Johnson et al., 2008; Mogg et al., 2015) who show abnormal distractor susceptibility (i.e., more errors/larger RTs in incongruent trials) in several paradigms, including the ANT version with arrow flankers as used here. Similarly, an attenuation of alerting states has been reported in ADHD (Johnson et al., 2008) and patients with strokes (Spaccavento et al., 2019) compared to the control population, but it was not seen in the amusic group. Finally, the age-related slowing in visual attention was similar in the amusic and control participants who were relatively old but age-matched suggesting that musical deficits have no distinctive impact on attentional performance with increasing age. Thus, the present study highlights preserved attention processing in congenital amusia, in keeping with the notion that their impairment is a selective musical disorder affecting pitch processing (Peretz et al., 2002; Peretz, 2016; Peretz and Vuvan, 2017).

Interestingly, finding normal visuo-spatial attention in congenital amusia does not support earlier claims of an intimate link between musical and visuo-spatial attentional processing abilities (Douglas and Bilkey, 2007), based on the assumption that sound frequency representations may be intertwined with spatial codes (e.g., lower pitch is usually represented lower in space than higher pitch) (Rusconi et al., 2006). Rather, the present study is more in line with prior studies showing preserved mental rotation abilities in amusics with low pitch or contour MBEA scores (Tillmann et al., 2010; Williamson et al., 2011). We found no attenuation of visuo-spatial attentional processing indices in amusic individuals, even in the most musically impaired, by showing no link between three distinct scores measuring musical deficits and attentional performance. These findings suggest that the severity of musical deficits does not impact visuo-spatial attention and further questions the notion of a continuum of visuo-spatial cognition with musical abilities (Douglas and Bilkey, 2007). However, we cannot disregard the possibility that more complex aspects of object representation/manipulation in space might interplay with musical skills, but any such interaction seems unrelated to the attentional processes probed here, again reinforcing the view that congenital amusia occurs independently of any other cognitive deficits.

### Effects of Emotional Music on Attention in Amusia

Our main goal with the present work was to assess whether the influence of music exposure on attentional processes has a similar effect on the congenital amusia population as compared to the general population. In accordance with previous work (Fernandez et al., 2020), we found a reliable modulation of processing speed, regardless of visual flanker congruency, when participants were exposed to joyful (i.e., high-arousing and high-valence) compared to sad music (i.e., low-arousing and low-valence), and this effect was similar in amusics and controls. This attentional enhancement was likely mainly driven by the arousal dimension of music since valence did not appear to modulate performance. Such effects of arousal might result from a greater engagement of the attentional control network in frontal and parietal cortices, as observed in a recent fMRI study using a similar paradigm (Fernandez et al., 2019b).

Unlike in previous work, however, we did not find significantly faster performances when comparing joyful musical exposure to the silence condition (Fernandez et al., 2019b), probably because of the small effect size of this modulation in the current population sample. However, the pattern of absolute RT effects (see Figure 3) fully accords with earlier observations, suggesting not only that joyful music produced fastest responses, while sad music and silence produced the slowest, but also that such differences reflect a facilitation of stimulus processing due to joyful (and more generally high-arousing) music rather than a slowing or distracting effect of (negative) emotional music relative to silence (Trost et al., 2014).

Overall, the normal influence of music exposure on executive attentional control in congenital amusia suggests that these individuals still receive the indirect effects of music in spite of their musical deficits. This finding supports the increasingly supported theory that an amusics' brain has the capacity to track subtle musical (pitch) variations without awareness (e.g., Peretz et al., 2009; Tillmann et al., 2012; Zendel et al., 2015). Taken together, our results highlight the powerful and pervasive capacity of music and musical emotions to influence the mind and our behavior through relatively automatic and unconscious pathways, including high-level cognitive functions associated with executive control. Our study yields precious insights into the remarkable relationships between emotional and attentional processing in the human brain through which music can enhance cognitive abilities.

A few possible limitations of the current study should be acknowledged. First of all, our main results and conclusions concerning the preserved attentional effects of musical emotions rely on negative findings, i.e., no significant differences between congenital amusics and controls in critical behavioral effects of interest. We feel that this finding is unlikely to be caused by insufficient power, given that our sample size was validated by previous work (Fernandez et al., 2019b). In addition, although the ANT has been successfully employed in several studies to assess major attentional components across various populations (Wang et al., 2005; Fernandez-Duque and Black, 2006; Mahoney et al., 2010; Park et al., 2019), it may have failed to capture another attentional dimension that is possibly affected by amusia. Nevertheless, the current finding of comparable visuo-spatial attentional performance in this paradigm, despite the congenital impairment associated with amusia, helps to further refine our understanding of this disorder. Further investigations should confirm intact attentional performances in amusics across a wider range of tasks, for instance, by comparing their performance to populations known to present attentional deficits. Finally, another general limitation of our work might be the relatively small sample of individuals with congenital amusia who were included in the study. As this condition has a low prevalence (1.5% up to 4%) in the general population, we deliberately chose strict inclusion criteria (i.e., scores below cut-off scores on two scale tests in accordance with the latest normative data) to ensure an inclusion of individuals presenting clear and substantial musical deficits only (Peretz and Vuvan, 2017; Vuvan et al., 2018), but this strictness inherently limited the size of our sample. We also acknowledge a potential lack of statistical power for some task conditions, particularly the alerting and orienting components whose assessment is made by computing a subset (half) of the total trials (see Fan et al., 2002), unlike the executive control component. This lack of statistical power might account for a failure to demonstrate a main effect of emotion on these components, contrary to our previous study (Fernandez et al., 2019b). In addition, the manipulation of the orienting components might have produced insufficient spatial preparation (50% validity contingency) and allowed participants to ignore the spatial cues, accounting for a lack of a significant validity effect during attentional orienting, unlike the original ANT paradigm (Fan et al., 2002).

In any case, to our knowledge, this study is the first to assess selective attention abilities as well as the influence of music exposure on attentional processes in congenital amusia. We were able to confirm normal emotional processing and cognitive control functions in amusics, notably by demonstrating that they exhibit similar accuracy and reaction time performances compared to the control population in the attentional network task measuring three distinct attentional components. Furthermore, they also exhibit faster reaction times in attention conflict conditions during joyful/high-arousing music compared to sad/low-arousing music, similar to people with normal music perception (Fernandez et al., 2019b). These data reveal that affect-related influences of music on attention control do not depend on the neural system altered in congenital amusia and still operate despite defective pitch processing. Our study yields insights on the remarkable relationships between emotional and attentional processing in the human brain through which music can enhance cognitive abilities.

## Data Availability Statement

The raw data supporting the conclusions of this article will be made available by the authors, without undue reservation.

## Ethics Statement

The studies involving human participants were reviewed and approved by Research Ethics Council for the Faculty of Arts and Sciences at the Université de Montréal. The patients/participants provided their written informed consent to participate in this study.

## Author Contributions

NF designed the project and validated the task. NF performed the data collection and analyses and wrote the initial draft of manuscript with contributions from other authors. PV supervised the research protocol, methodology, and analyses. NG and IP supervised the research protocol and provided testing material and resources for data collection. All authors actively contributed to the revision, corrections of the manuscript, approved the final version for publication, and contributed to the research question formulation.

## Conflict of Interest

The authors declare that the research was conducted in the absence of any commercial or financial relationships that could be construed as a potential conflict of interest.
